# Comparison study of quantitative susceptibility mapping with GRAPPA and wave-CAIPI: reproducibility, consistency, and microbleeds detection

**DOI:** 10.1007/s11604-024-01683-4

**Published:** 2024-10-29

**Authors:** Azusa Sakurama, Yasutaka Fushimi, Satoshi Nakajima, Akihiko Sakata, Sachi Okuchi, Takayuki Yamamoto, Sayo Otani, Krishna Pandu Wicaksono, Satoshi Ikeda, Shuichi Ito, Takakuni Maki, Wei Liu, Yuji Nakamoto

**Affiliations:** 1 Department of Diagnostic Imaging and Nuclear Medicine, Kyoto University Graduate School of Medicine, 54 Shogoin Kawahara-Cho, Sakyo-Ku, Kyoto, 606-8507 Japan; 2 Department of Radiology, Faculty of Medicine, Universitas Indonesia–Dr. Cipto Mangunkusumo National Central General Hospital, Jakarta, Indonesia; 3 Department of Neurology, Kyoto University Graduate School of Medicine, Kyoto, Japan; 4 Siemens Shenzhen Magnetic Resonance Ltd, Shenzhen, China

**Keywords:** Quantitative susceptibility mapping, Wave-CAIPI, Generalized autocalibrating partially parallel acquisition, Microbleeds

## Abstract

**Purpose:**

We compared quantitative susceptibility mapping (QSM) with wave-CAIPI 9 × (QSM_WC9 ×) with reference standard QSM with GRAPPA 2 × (QSM_G2 ×) in two MR scanners. We also compared detectability of microbleeds in both QSMs to demonstrate clinical feasibility of both QSMs.

**Materials and methods:**

This prospective study was approved by the institutional review board and written informed consent was obtained from each subject. Healthy subjects were recruited to evaluate intra-scanner reproducibility, inter-scanner consistency, and inter-sequence consistency of QSM_G2 × and QSM_WC9 × at 2 MR scanners. Susceptibility values measured with volume of interests (VOIs) were evaluated. Patients who were requested for susceptibility weighted imaging were also recruited in this study to measure microbleeds on QSM_G2 × and QSM_WC9 × . The number of microbleeds was compared between two QSMs.

**Results:**

Total 55 healthy subjects (male 34, female 21, 38.3 years [23–79]) were included in this study. We investigated reproducibility and consistency of QSM_WC9 × by comparing reference standard QSM_G2 × in two MR scanners in this study, and high correlation (ρ, 0.93–0.97) and high intraclass correlation coefficient (ICC) (0.97–0.99) were obtained. Sixty patients (male 30, female 30; age, 55.4 years [21–85]) were finally enrolled in this prospective study. The ICC of the detected number of microbleeds between QSM_G2 × and QSM_WC9 × was 0.99 (0.98–0.99).

**Conclusion:**

QSM_WC9 × and reference standard QSM_G2 × in two MR scanners showed good reproducibility and consistency in estimating magnetic susceptibilities. QSM_WC9 × and QSM_G2 × were also comparable in terms of microbleeds detection with good agreement of raters and high ICC.

**Supplementary Information:**

The online version contains supplementary material available at 10.1007/s11604-024-01683-4.

## Introduction

Quantitative susceptibility mapping (QSM) is a post-processing technique to quantitatively estimate local magnetic susceptibility in biologic tissue using gradient-recalled echo (GRE)-phase measurement [1–4]. QSM provides quantitative information on magnetic susceptibility which is useful for differentiating between paramagnetic and diamagnetic susceptibility. QSM has proven to be a valuable tool for assessing the deposition of paramagnetic substances, such as iron deposition, hemosiderin, gadolinium, and diamagnetic substances, such as calcification and myelin [5–8]. QSM has thus been applied for evaluating various conditions, such as microbleeds [9, 10], iron accumulation associated with aging changes [11–13], gadolinium deposition [14, 15], and neurodegeneration such as Parkinson’s disease [16, 17]. Although the usefulness of QSM is well established, QSM has not yet seen common use in clinical practice, partly because of the long scan time [18].

Parallel imaging techniques such as generalized autocalibrating partially parallel acquisition (GRAPPA) and sensitivity encoding (SENSE) have been introduced to reduce scan time and preserve signal-to-noise ratio (SNR) [19, 20]. By taking advantage of coil sensitivity encoding from multi-channel receiver coils, GRE with an acceleration factor of 2 is generally applied for QSM with parallel imaging techniques such as SENSE and GRAPPA, but the ability to reduce scan time for QSM with GRE remains limited. Using other sequences such as 3D echo planar imaging (EPI), scan times for QSM data can be reduced to around 2 min [9]. In a multi-shot approach with short EPI train lengths, distortion and blurring become limited, and images with a considerable gain in both SNR and coverage as compared to GRE imaging can be obtained within a given amount of scan time [21]. The disadvantage of 3D EPI is the data acquisition at only one echo time. On the other hand, higher acceleration for QSM with GRE has not been evaluated in detail. Recently, wave-controlled aliasing in parallel imaging (CAIPI) acquisition enables highly accelerated volumetric imaging with fewer artifacts and low SNR penalties by playing sinusoidal gradients during the readout of each phase encoding line [22–29]. The acceleration factor for wave-CAIPI has been reported as ninefold at most (threefold for phase encoding, threefold for slice encoding).

More acceleration for GRE has been expected to create QSM using wave-CAIPI, and several reports have described susceptibility-weighted imaging (SWI) with wave-CAIPI [30, 31]. Using wave-CAIPI, higher spatial resolution than the previous 3D EPI report can be achieved at 3 T with a scan time less than 2 min [21, 31]. However, QSM with wave-CAIPI has not been evaluated in detail in clinical practice [32]. We hypothesized that QSM with ninefold acceleration using wave-CAIPI could be used reliably in clinical scanners in terms of reproducibility and consistency. The purpose of this study was to compare QSM with wave-CAIPI 9 × (QSM_WC9 ×) with the reference standard QSM with GRAPPA 2 × (QSM_G2 ×) in two MR scanners. We also compared the detectability of microbleeds in both QSMs to demonstrate clinical feasibility of both QSMs.

### Materials and methods

This prospective study was performed in accordance with the Declaration of Helsinki and was approved by Kyoto University Graduate School and Faculty of Medicine, Ethics Committee. Written informed consent was obtained from each subject.

### Healthy subjects

**Fig. 1:**
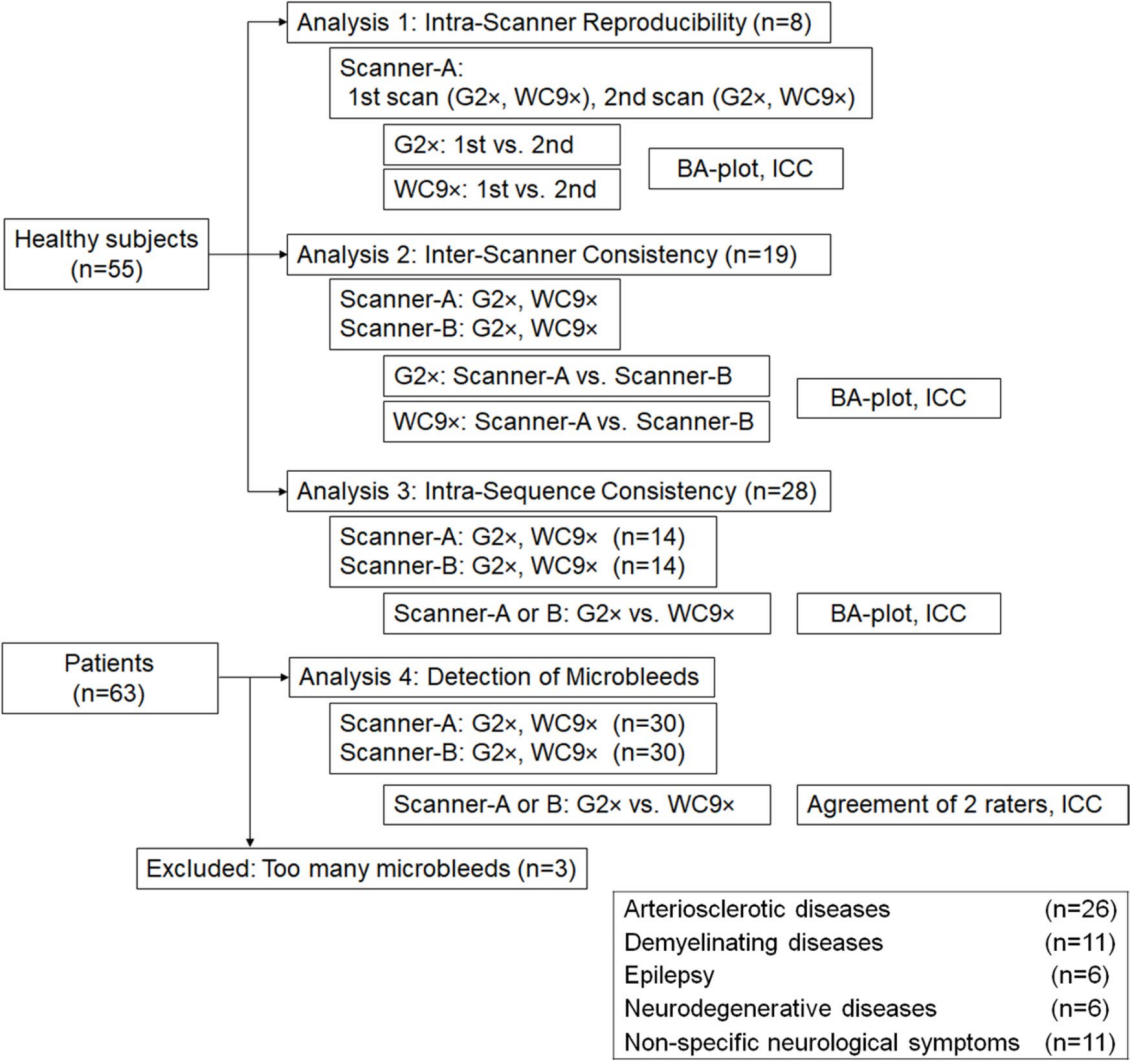
Enrollment of healthy subjects and patients

Healthy subjects were recruited to evaluate: (1) intra-scanner reproducibility; (2) inter-scanner consistency; and (3) inter-sequence consistency of QSM_G2 × and QSM_WC9 × . Exclusion criteria for these healthy subjects were as follows: (1) severe head motion during imaging; or (2) incidental findings such as cerebral infarction, old hemorrhage, and other abnormalities. In total, 55 healthy subjects (34 men, 21 women; mean age, 38.3 years; range, 23–79 years) were included in this study (Fig. 1).

#### Analysis 1, intra-scanner reproducibility (single scanner, test and re-test) (*n* = 8)

Intra-scanner reproducibility was analyzed with the test and re-test data of a subject who underwent two scans with a single MR unit (Scanner A). QSM_G2 × (1st scan, 2nd scan), and QSM_WC9 × (1st scan, 2nd scan) were evaluated, respectively (*n* = 8). Note that head position differed between 1st scan and 2nd scan.

#### Analysis 2, inter-scanner consistency (different scanners, same sequence) (*n* = 19)

Inter-scanner consistency was analyzed using data from subjects who underwent scans with 2 MR units (Scanners A and B) (*n* = 19). QSM_G2 × (Scanner A and B) and QSM_WC9 × (Scanner A and B) were both evaluated. Note that head positions differed, since the data were obtained from different scanners.

#### Analysis 3, inter-sequence consistency (same scanner, different sequences) (*n* = 28)

Subjects (*n* = 28) underwent MR scans with both Scanner A (*n* = 14) and Scanner B (*n* = 14). QSM_G2 × and QSM_WC9 × were evaluated with Scanner A. QSM_G2 × and QSM_WC9 × were also evaluated with Scanner B. Note that head position did not change during the two sequences.

These subjects also underwent GRE with GRAPPA 9 × and GRE with CAIPI 9 × , then QSM_G9 × and QSM_C9 × were created, respectively. However, these data were not included for analyses due to severe parallel imaging artifacts that were always present (representative figures with QSM_G9 × and QSM_C9 × are shown in Supplemental Fig. 1).

### Patients

Patients for whom SWI was requested between July 2019 and August 2020 were also recruited in this study to measure microbleeds on QSM_G2 × and QSM_WC9 × . Exclusion criteria for these patients were as follows: 1) severe head motion; or 2) more than 30 microbleeds. Three patients with brain contusion (*n* = 2) and cerebral amyloid angiopathy (*n* = 1) were excluded due to too many microbleeds. Sixty patients (30 women, 30 men; age, 55.4 years; range, 21–85 years) were finally enrolled in this prospective study (Fig. 1).

### MR imaging

MR imaging was performed with two 3-T MR scanners (MAGNETOM Prisma; Siemens Healthineers, Erlangen, Germany) with a 64-channel head/neck coil in our institute (Scanners A and B). Imaging with 3D gradient echo (GRE) sequences was performed using the following parameters in common: TR, 32 ms; TE, 20 ms; flip angle, 15°; bandwidth, 80 Hz/pixel; field of view, 230 × 230 mm^2^; matrix, 320 × 294; spatial resolution, 0.72 × 0.72 × 1.0 mm^3^. For acceleration, 24 reference lines were acquired in the phase-encoding direction for all GREs, and reference lines in slice direction (and scan time) were as follows: (1) GRE with GRAPPA 2 × 1 (GRE_G2 ×), not accelerated in slice direction (6 min 8 s); (2) GRE with a prototype wave-CAIPI 3 × 3 (GRE_WC9 ×), using 24 reference lines in slice direction with a CAIPI shift factor of 1 (1 min 25 s).

Images were also obtained using 3D T1-magnetization prepared rapid acquisition gradient-echo (MPRAGE), with: TR, 2300 ms; TE, 4.67 ms; flip angle, 9°; and bandwidth, 130 Hz/pixel. Spatial resolution was isotropic voxel of 0.9 mm, and 208 slices were acquired. For acceleration, 24 reference lines were acquired in the phase-encoding direction for all MPRAGEs (GRAPPA 2 × 1), and not accelerated in slice direction. Scan time was 5 min 21 s.

### Post-imaging analysis for healthy subjects

**Fig. 2:**
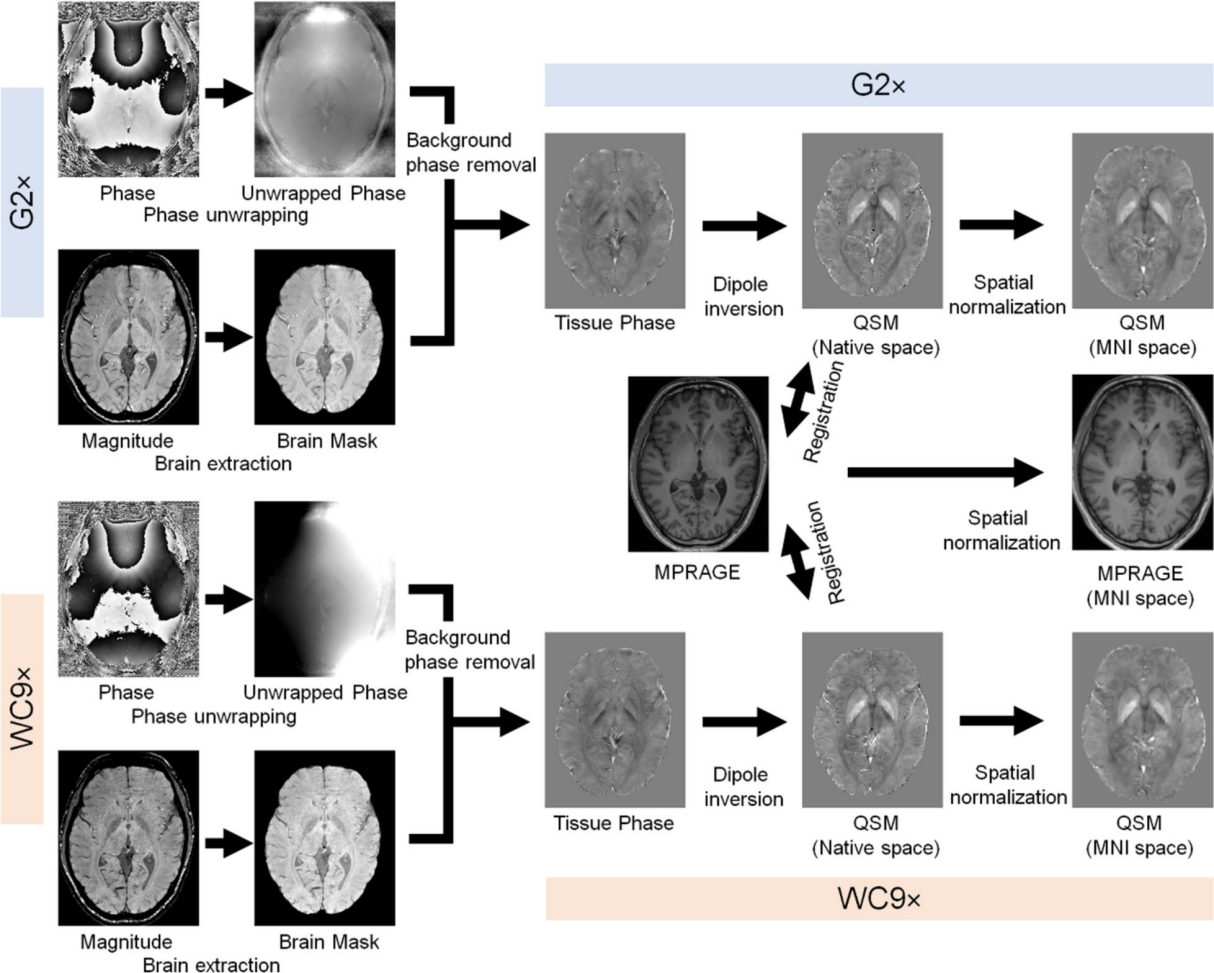
Post-imaging analysis for healthy subjects. QSM was created from magnitude and phase images of corresponding 3D GRE (G2 × and WC9 ×). After performing Laplacian-based phase unwrapping, variable-kernel sophisticated harmonic artifact reduction for phase data, and dipole inversion, QSM was created. Next, 3D-MPRAGE images were registered to 3D GRE images of the corresponding magnitude. Registered 3D-MPRAGE and QSM were normalized to the MNI space

QSM was created from magnitude and phase images of 3D GRE using STI Suite version 3 (https://people.eecs.berkeley.edu/~chunlei.liu/software) (Fig. 2). Laplacian-based phase unwrapping, variable-kernel sophisticated harmonic artifact reduction for phase data, and dipole inversion were performed, and then, QSM was created [4]. The 3D-MPRAGE images were registered to 3D GRE images of the corresponding magnitude. Registered 3D-MPRAGE images were segmented to create a Diffeomorphic Anatomical Registration Through Exponentiated Lie Algebra (DARTEL) template using SPM12 (https://www.fil.ion.ucl.ac.uk/spm/software/spm12/). This template was used to normalize QSM to the MNI space and perform average QSM among patients.

The following volumes of interest (VOIs) were then applied to QSM: splenium of the corpus callosum, caudate nucleus, putamen, optic radiation, internal capsule, globus pallidus, substantia nigra, red nucleus, and dentate nucleus (Supplemental Fig. 2).

### Evaluation of microbleeds in patients

Patients underwent QSM_G2 × and QSM_WC9 × in either of the two MR units. Microbleeds were evaluated by two raters (A.S. and K.P.W., each with 9 years of experience in neuroradiology). If the raters detected microbleeds, the number of microbleeds was compared. In the case of discrepancies between the two raters, the number of microbleeds was determined by another radiologist (Y.F., with 25 years of experience in neuroradiology).

### Calculation of SNR

Calculation of SNR was performed by serial measurements of GRE sequences for the spherical phantom (NiSO_4_·6H_2_O). Specifically, GRE_G2 × , GRE_WC9 × , GRE_C9 × , and GRE_G9 × were measured 10 times and the SNR map was created with the mean value divided by the standard deviation.

### Statistical analysis

Intraclass correlation coefficient (ICC) and 95% confidence intervals (CIs) of susceptibility values were calculated. Bland–Altman analysis was performed for susceptibility values. The inter-observer agreement for detection of microbleeds was assessed with ICC.

Statistical analysis was performed using MedCalc version 18 (MedCalc Software, Ostend, Belgium) and JMP Pro version 16.0 (SAS Institute Inc., Cary, NC, United States).

## Results

### Healthy subjects

**Fig. 3:**
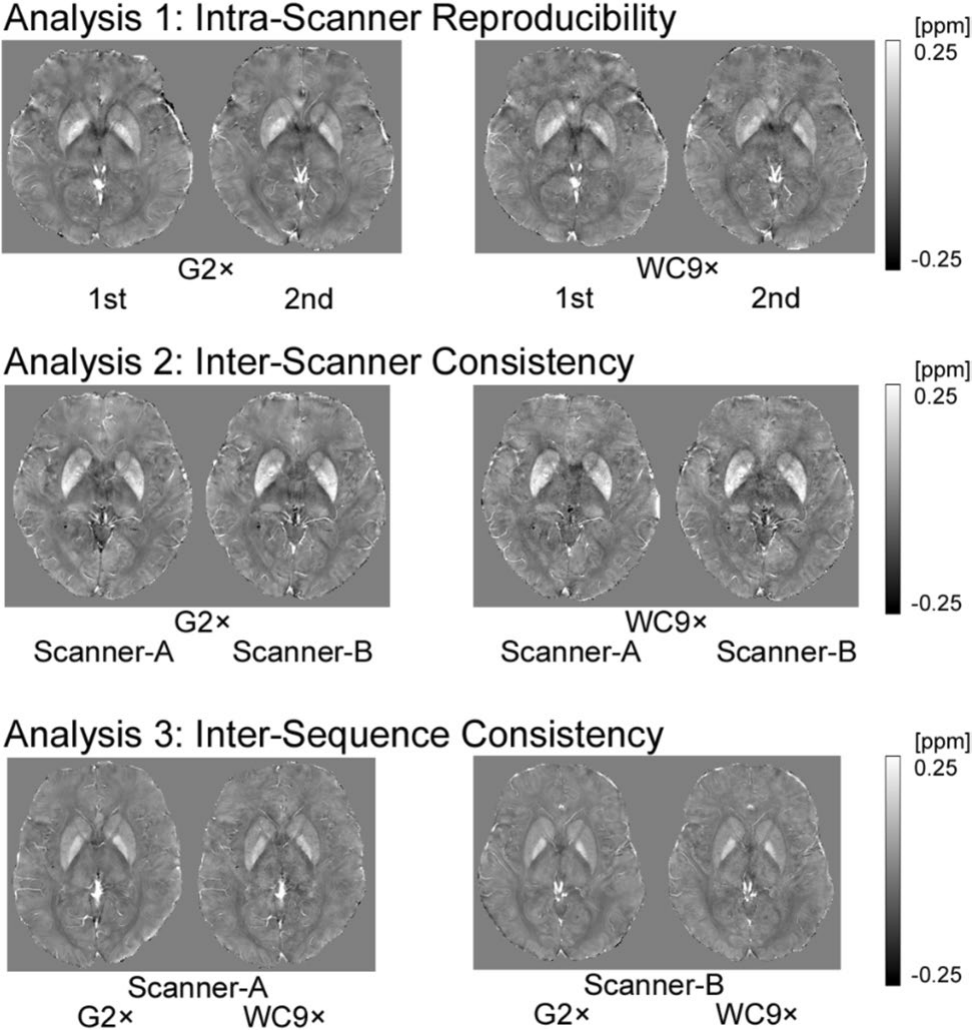
Representative images of healthy subjects evaluated in Analysis 1, Intra-scanner reproducibility (single scanner, test and re-test), Analysis 2, Inter-scanner consistency (different scanners, same sequence), and Analysis 3, Inter-sequence consistency (same scanner, different sequences)

Representative images of each analysis are shown in Fig. 3.

#### Analysis 1, intra-scanner reproducibility (single scanner, test and re-test)

**Fig. 4:**
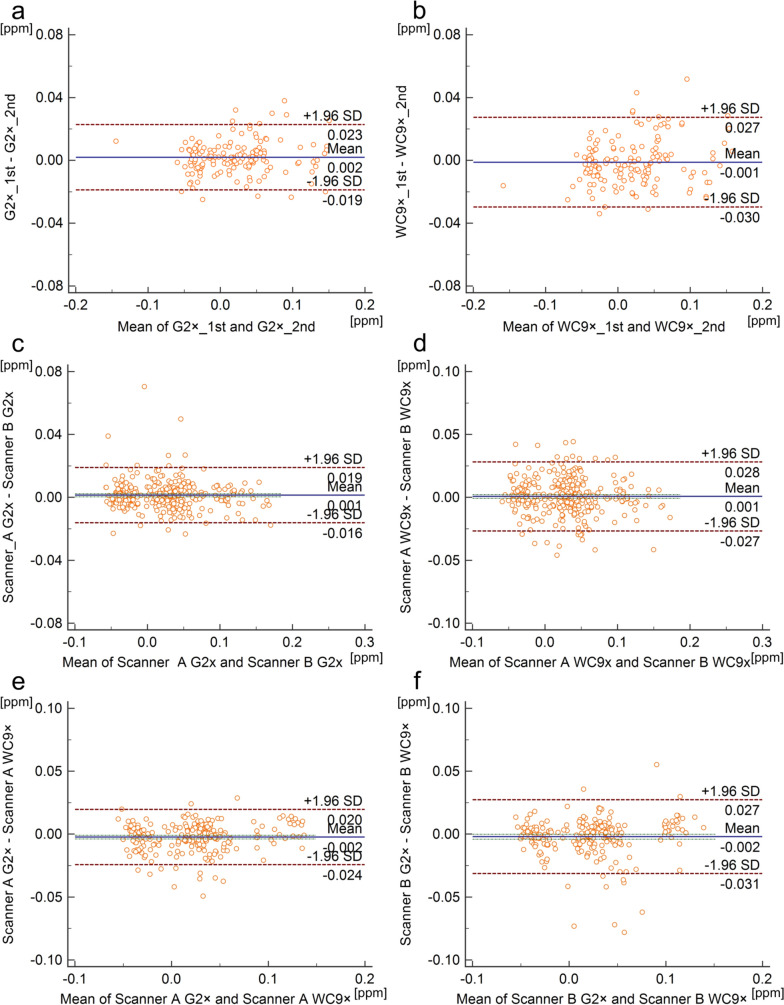
Bland–Altman plots of Analysis 1 **a**, **b**, Analysis 2 **c**, **d**, and Analysis 3 **e**, **f**. Details of each analysis are as follows: Analysis 1, susceptibility values between 1st scan and 2nd scan in QSM_G2 × **a** and QSM_WC9 × **b**; Analysis 2, **s**usceptibility values of QSM_G2 × **c** and QSM_WC9 × **d** obtained from Scanners A and B; and Analysis 3, QSM_G2 × and QSM_WC9 × from Scanner A **e**, and QSM_G2 × and QSM_WC9 × from Scanner B **f**

The ICC of QSM_G2 × (1st scan, 2nd scan) was a median of 0.98 (interquartile range, 0.97–0.99), and the ICC of QSM_WC9 × (1st scan, 2nd scan) was 0.97 (0.96–0.98) (Table 1). Bland–Altman plots showed strong agreement between QSM_G2 × (1st scan, 2nd scan), with a mean difference of 0.002 ppm (95% confidential interval (CI), −0.019 to 0.023 ppm), and QSM_WC9 × (1st scan, 2nd scan), with a mean difference of −0.001 ppm (95%CI −0.030 to 0.027 ppm) (Fig. 4).

**Table 1:** Results of Analysis 1, Intra-scanner reproducibility; Analysis 2, Inter-scanner consistency; and Analysis 3, Inter-sequence consistency

Analysis 1	ICC
QSM_G2 × 1st scan vs. QSM_G2 × 2nd scan	0.98 [0.97–0.99]
QSM_WC9 × 1st scan vs. QSM_WC9 × 2nd scan	0.97 [0.96–0.98]
Analysis 2	ICC
Scanner-A QSM_G2 × vs. Scanner-B QSM_G2 ×	0.99 [0.99–0.99]
Scanner-A QSM_WC9 × vs. Scanner-B QSM_WC9 ×	0.98 [0.97–0.98]
Analysis 3	ICC
Scanner-A QSM_G2 × vs. Scanner-A QSM_WC9 ×	0.98 [0.98–0.99]
Scanner-B QSM_G2 × vs. Scanner-B QSM_WC9 ×	0.97 [0.96–0.98]

#### Analysis 2, inter-scanner consistency (different scanners, same sequence)

The ICC of QSM_G2 × obtained at Scanner A and B was 0.99 (0.99–0.99), and the ICC of QSM_WC9 × (Scanner A and B) was 0.98 (0.97–0.98) (Table 1). Bland–Altman plots showed strong agreement between QSM_G2 × (Scanner A and B), with a mean difference of 0.001 ppm (95%CI -0.016 to 0.019 ppm) and QSM_WC9 × (Scanner A and B), with a mean difference of 0.001 ppm (95%CI −0.027 to 0.028 ppm) (Fig. 4).

#### Analysis 3, inter-sequence consistency (same scanner, different sequences)

The ICC of QSM_G2 × and QSM_WC9 × at Scanner A was 0.98 (0.98–0.99), and the ICC of QSM_G2 × and QSM_WC9 × at Scanner B was 0.97 (0.96–0.98) (Table 1). Bland–Altman plots showed strong agreement between QSM_G2 × and QSM_WC9 × at Scanner A, with a mean difference of −0.002 ppm (95%CI -0.024 to 0.020 ppm), and between QSM_G2 × and QSM_WC9 × at Scanner B, with a mean difference of −0.002 ppm (95%CI −0.031 to 0.027 ppm) (Fig. 4).

Susceptibility values for each VOI in Analyses 1, 2 and 3 are shown in Table 2.

**Table 2:** Susceptibility values of each VOI in Analyses 1, 2, and 3

Analysis 1		DN	SN	RN	PT	GP	CN	OR	IC	Splenium
QSM_G2 × [ppm]	1st scan	0.03 ± 0.02	0.05 ± 0.03	0.02 ± 0.03	0.05 ± 0.02	0.13 ± 0.02	0.05 ± 0.01	− 0.03 ± 0.01	− 0.04 ± 0.01	− 0.02 ± 0.01
QSM_G2 × [ppm]	2nd scan	0.02 ± 0.02	0.04 ± 0.02	0.01 ± 0.02	0.05 ± 0.02	0.13 ± 0.01	0.05 ± 0.01	− 0.03 ± 0.01	− 0.04 ± 0.01	− 0.02 ± 0.01
QSM_WC9 × [ppm]	1st scan	0.02 ± 0.02	0.05 ± 0.03	0.01 ± 0.03	0.05 ± 0.02	0.12 ± 0.03	0.05 ± 0.02	− 0.04 ± 0.01	− 0.04 ± 0.01	− 0.02 ± 0.01
QSM_WC9 × [ppm]	2nd scan	0.02 ± 0.03	0.04 ± 0.03	0.01 ± 0.03	0.05 ± 0.02	0.12 ± 0.02	0.05 ± 0.02	− 0.04 ± 0.01	− 0.04 ± 0.01	− 0.02 ± 0.01

Analysis 2		DN	SN	RN	PT	GP	CN	OR	IC	Splenium
QSM_G2 × [ppm]	Scanner A	0.06 ± 0.03	0.09 ± 0.02	0.08 ± 0.02	0.06 ± 0.02	0.14 ± 0.03	0.04 ± 0.02	0.03 ± 0.01	− 0.04 ± 0.02	− 0.02 ± 0.01
QSM_G2 × [ppm]	Scanner B	0.06 ± 0.03	0.09 ± 0.02	0.07 ± 0.03	0.06 ± 0.02	0.14 ± 0.03	0.04 ± 0.02	− 0.03 ± 0.01	-0.04 ± 0.02	− 0.02 ± 0.01
QSM_WC9 × [ppm]	Scanner A	0.06 ± 0.03	0.09 ± 0.02	0.08 ± 0.02	0.06 ± 0.02	0.14 ± 0.03	0.04 ± 0.02	− 0.03 ± 0.01	− 0.04 ± 0.02	− 0.02 ± 0.01
QSM_WC9 × [ppm]	Scanner B	0.06 ± 0.02	0.09 ± 0.02	0.08 ± 0.03	0.05 ± 0.02	0.14 ± 0.03	0.04 ± 0.02	− 0.03 ± 0.01	− 0.02 ± 0.03	− 0.01 ± 0.01

Analysis 3	DN	SN	RN	PT	GP	CN	OR	IC	Splenium
Scanner A QSM_G2 × [ppm]	0.02 ± 0.01	0.04 ± 0.01	0.01 ± 0.01	0.03 ± 0.01	0.11 ± 0.02	0.04 ± 0.01	0.03 ± 0.01	− 0.04 ± 0.01	− 0.02 ± 0.01
Scanner A QSM_WC9 × [ppm]	0.02 ± 0.01	0.05 ± 0.02	0.03 ± 0.02	0.03 ± 0.01	0.11 ± 0.02	0.04 ± 0.01	− 0.03 ± 0.01	− 0.04 ± 0.01	− 0.01 ± 0.02
Scanner B QSM_G2 × [ppm]	0.02 ± 0.01	0.04 ± 0.01	0.02 ± 0.01	0.03 ± 0.01	0.12 ± 0.01	0.04 ± 0.00	− 0.04 ± 0.01	− 0.03 ± 0.01	− 0.02 ± 0.01
Scanner B QSM_WC9 × [ppm]	0.02 ± 0.02	0.05 ± 0.02	0.03 ± 0.02	0.03 ± 0.01	0.11 ± 0.02	0.04 ± 0.01	− 0.04 ± 0.01	− 0.03 ± 0.02	− 0.02 ± 0.02

DN, dentate nucleus; SN, substantia nigra; RN, red nucleus; PT, putamen; GP, globus pallidus; CN, caudate nucleus; OR, optic radiation; IC, internal capsule; Splenium, splenium of the corpus callosum.

Details of healthy subjects included for each analysis are as follows: Analysis 1, 62.8 years [67.5, 38–79], 5 males, 3 females; Analysis 2, 46.2 years [40, 23–73], 11 males, 8 females; Analysis 3, 25.9 years [26, 23–36], 18 males, 10 females.

### Evaluation of microbleeds in patients

Both QSM_G2 × and QSM_WC9 × were obtained from the MR scans of 60 patients (32 females, 28 males; 55.1 ± 18.5 years). Demographics of the patients are shown in Fig. 1. In QSM_G2 × , Raters A and B detected 116 and 120 microbleeds, respectively, while Raters A and B detected 108 and 122 microbleeds in QSM_WC9 × , respectively. Good agreement was observed between the two raters, with an ICC for QSM_G2 × of 0.81 (0.70–0.88), and for QSM_WC9 × of 0.89 (0.80–0.93); disagreement between 2 raters was 13 out of 60, and 11 out of 60, respectively.

**Fig. 5:**
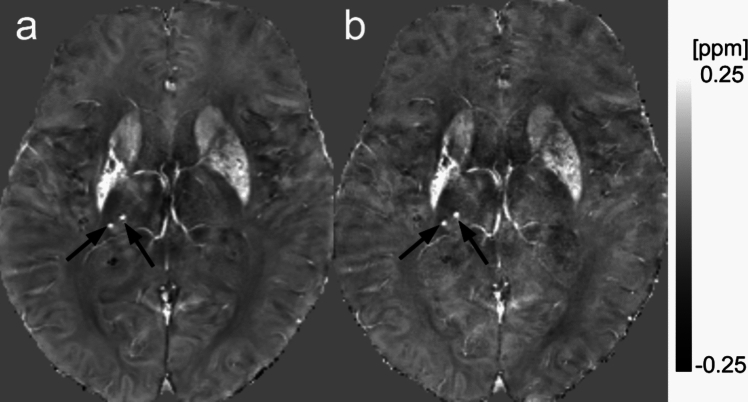
Representative images with microbleeds. A 77-year-old woman with old putaminal infarction. Both QSM_G2 × **a** and QSM_WC9 × **b** show high susceptibility spots suggesting microbleeds in the right thalamus (arrows)

The final number of microbleeds determined by another neuroradiologist in the case of discrepancies between the two raters showed: QSM_G2 × , mean, 1.6 (median, 0, range, 0–26); QSM_WC9 × , mean, 1.6 (median 0, range 0–23). The ICC of the detected number of microbleeds between QSM_G2 × and QSM_WC9 × was 0.99 (0.98–0.99). Representative images of QSM_G2 × and QSM_WC9 × with microbleeds are shown in Fig. 5.

### SNR map

SNR of GRE_G2 × , GRE_WC9 × , GRE_C9 × , and GRE_G9 × were 221.6 ± 90.0, 158.2 ± 71.7, 156.4 ± 75.9, and 80.0 ± 47.1. SNR of GRE_WC9 × was the second best among 4 GRE sequences, and considering that the scan time was reduced to 1 min 25 s versus 6 min 8 s, SNR of GRE_WC9 × was better maintained. SNR maps of each GRE image are shown in Supplemental Fig. 3. There was not much difference of SNR between WC9 × and C9 × . The FOV was large relative to the phantom, and the N/2 artifact did not overlap the phantom, which may have the reason of relatively less SNR reduction in C9 × .

## Discussion

We investigated the reproducibility and consistency of QSM_WC9 × by comparing reference standard QSM_G2 × in two MR scanners in this study, obtaining a high ICC (0.97–0.99). Detection of microbleeds was also compared between the two QSMs, and good agreement was observed between the two raters (ICC, 0.99).

We demonstrated high reproducibility and consistency of QSM_WC9 × compared with QSM_G2 × by evaluating VOIs in healthy subjects at clinical 3 T scanners. Although the reproducibility of QSM using wave-CAIPI at 3 T has not previously been evaluated in detail, QSM at 7 T with 0.5-mm isotropic resolution nonlinear dipole inversion conducted using wave-CAIPI encoding (15 ×) was reported as feasible [32]. Using a newly developed acceleration technique (joint virtual coil GRAPPA), QSM_9 × was created in 2 min, making it feasible for clinical use [33]. Our results may become the reference of future studies for accelerated QSM sequences.

A high ICC for the detected number of microbleeds between QSM_G2 × and QSM_WC9 × was observed in this study, consistent with the results of a previous paper on SWI WC6 × within half of the scan time of SWI G2 × , showing high agreement for microbleed detection and diagnosis of intracranial lesions compared to SWI G2 × [31]. The scan time for wave-CAIPI in this study was shorter than that of the previous study, which may have been beneficial for uncooperative patients at risk of motion artifacts [31].

Several limitations should be noted. First, the generalizability of our findings was constrained by the sample size and the limited pathology of microbleeds. Future studies with a larger sample size are essential to validate and extend our results, and QSM_WC9 × should be applied to a broader spectrum of pathologies. Second, wave-CAIPI required time to process images and calculation of QSM was performed off-console. Recent continuous advances in image processing hardware have reduced the image reconstruction of wave-CAIPI, which may facilitate the clinical application of QSM_WC9 × . Third, the age distribution of healthy subjects differed among Analyses 1, 2, and 3, which may lead to differences in mean susceptibility values among analyses. Fourth, discrepancies were observed between the two raters in the detection of microbleeds, partly due to a total miscount in a large number of microbleed cases or a simple oversight, but no certain trend could be noted. Despite this limitation, magnetic susceptibility information can be retained even at 9 × acceleration using wave-CAIPI, facilitating QSM without concern for imaging time.

In conclusion, QSM_WC9 × and reference standard QSM_G2 × in two MR scanners showed good reproducibility and consistency for estimating magnetic susceptibilities. Detectability of microbleeds on QSM_WC9 × and QSM_G2 × was in good agreement with raters and showing high ICC, suggesting that QSM_WC9 × can be used as a substitute for QSM_G2 × .

## Supplementary Information

Below is the link to the electronic supplementary material.

Supplementary file1 (TIF 4720 kb)

Supplementary file2 (TIF 641 kb)

Supplementary file3 (TIF 6131 kb)
