# Supplementary figures and images for: Comparison study of quantitative susceptibility mapping with GRAPPA and wave-CAIPI: reproducibility, consistency, and microbleeds detection
Source: Jpn J Radiol. 2024 Oct 29;43(3):379–88. doi: 10.1007/s11604-024-01683-4 (PMC11868234; doi:10.1007/s11604-024-01683-4)

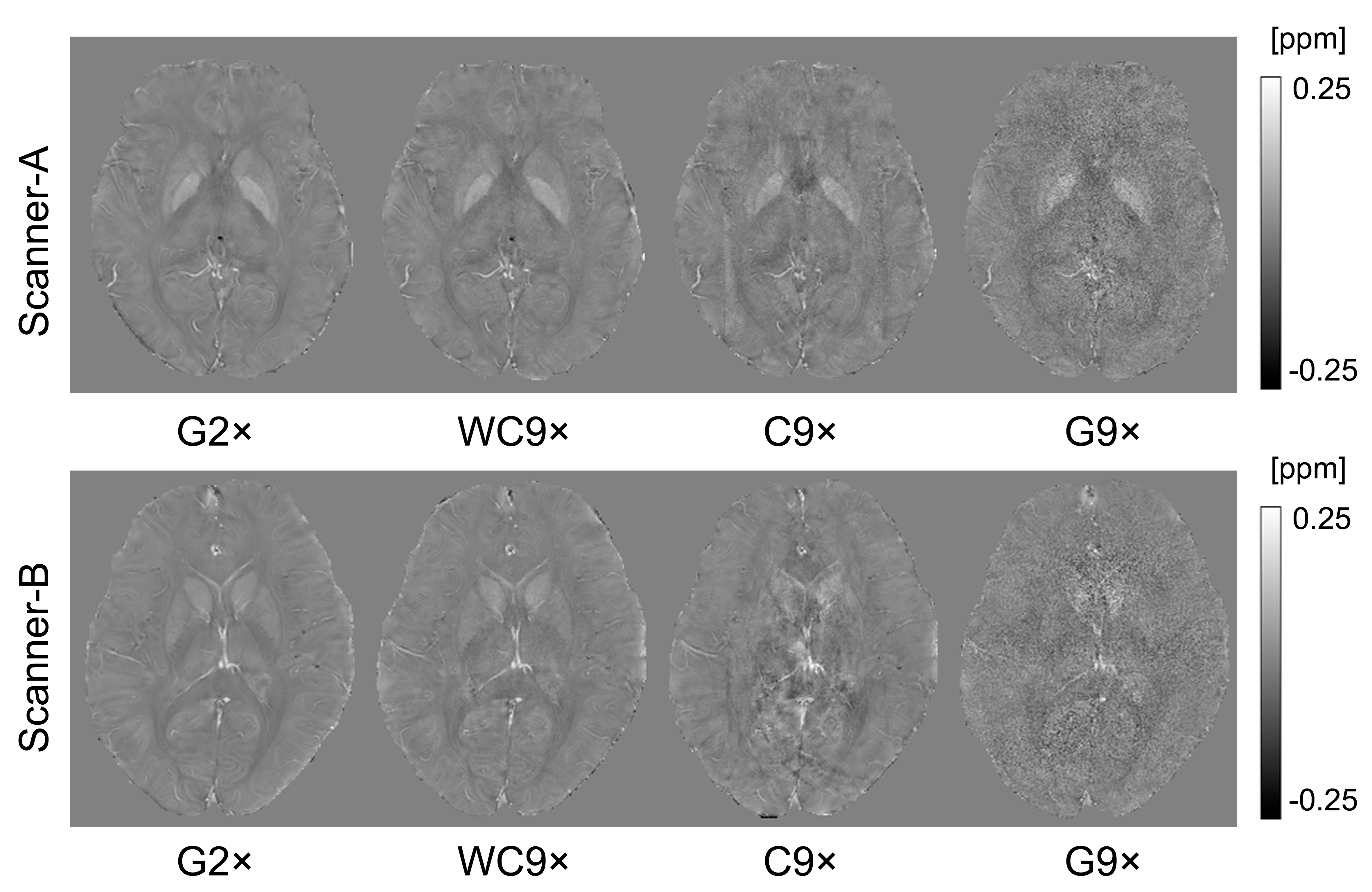

Supplement: Supplementary file 1 — Supplementary file1 (TIF 4720 kb) [file 11604_2024_1683_MOESM1_ESM.tif]

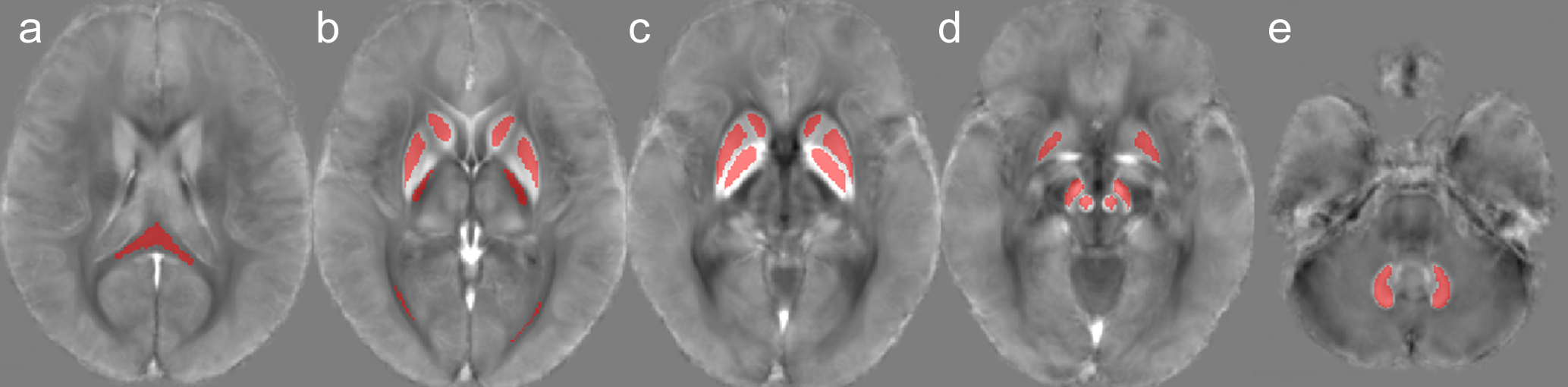

Supplement: Supplementary file 2 — Supplementary file2 (TIF 641 kb) [file 11604_2024_1683_MOESM2_ESM.tif]

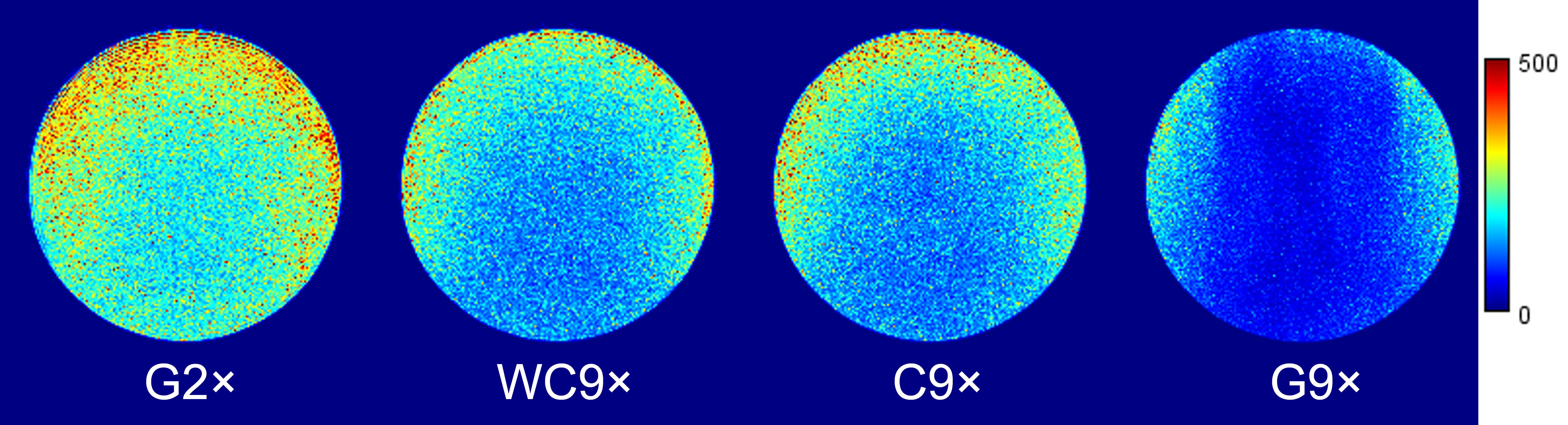

Supplement: Supplementary file 3 — Supplementary file3 (TIF 6131 kb) [file 11604_2024_1683_MOESM3_ESM.tif]
